# The qSOFA requires validation as a promptly applicable clinical criterion

**DOI:** 10.1002/ams2.287

**Published:** 2017-07-20

**Authors:** Shigeki Kushimoto, Satoshi Gando, Hiroshi Ogura

**Affiliations:** ^1^ Division of Emergency and Critical Care Medicine Tohoku University Graduate School of Medicine Sendai Miyagi Japan; ^2^ Division of Acute and Critical Care Medicine Department of Anesthesiology and Critical Care Medicine Hokkaido University Graduate School of Medicine Hokkaido Japan; ^3^ Department of Traumatology and Acute Critical Medicine Osaka University Graduate School of Medicine Osaka Japan

Along with the new conceptual definition for sepsis, the quick Sequential Organ Failure Assessment (qSOFA) score was proposed as a simple clinical criterion to potentially assist clinicians in identifying patients with suspected infection who are likely to have a prolonged intensive care unit (ICU) stay or die in the hospital, which can be identified immediately at the bedside.^1^, ^2^ The qSOFA can be estimated promptly and repeatedly without laboratory tests. It was proposed as a clinical criterion, that is, positive or negative, not a scoring system.^1^

In the recent study published in JAMA: The Journal of the American Medical Association, Raith and colleagues^3^ reported the results of external validation of criteria for sepsis using a large database. They assessed the discriminatory capacities of an increase in SOFA score by two or more points, the systemic inflammatory response syndrome (SIRS) criteria, and the qSOFA measured within the first 24 h of admission for predicting the outcomes of critically ill patients with suspected infection.

However, the authors retrospectively evaluated the individual components of qSOFA and SIRS,^4^ using the worst values obtained within 24 h after ICU admission from the Australian and New Zealand Intensive Care Society Database, as scoring systems. Even though the qSOFA was proposed as a screening tool that could achieve prompt results at the bedside,^1^ the worst values obtained at different time points within 24 h of ICU admission were independently selected, and component variables were used individually.^3^ Their results did not reflect the discriminatory capacities of the qSOFA and SIRS criteria,^4^ and the study design was not appropriate for external validation of the qSOFA, as a proposed criterion which could be promptly applicable at the bedside.

Raith and colleagues retrospectively evaluated the qSOFA and SIRS using the worst values obtained within 24 h after ICU admission as assessing the Acute Physiology and Chronic Health Evaluation (APACHE) scores. Therefore, we would like to also ask why the authors did not use the APACHE scores to compare the prognostic accuracy of patients with suspected infection admitted to the ICU. The APACHE scores use the worst values 24 h after admission to the ICU, which was proposed as a prognostic scoring system of disease severity that are used to predict outcomes, typically mortality, in patients admitted to ICU. The APACHE scores would be more suitable to predict the outcome of the ICU patients. We believe that the time of diagnosis is the most crucial aspect when diagnosing sepsis. Using the worst values obtained during the first 24 h does not affect early sepsis diagnosis.

Based on these issues the title and conclusions of the study may be misleading. A prospective study with a well‐designed protocol is required to validate qSOFA as a promptly and repeatedly applicable criterion for identifying patients with suspected infection who are likely to have a prolonged ICU stay or die in the hospital, in which the comparison with SIRS and other variables for screening must be included. The Japanese Association of Acute Medicine multicenter study group is preparing a prospective study, and we believe that the validation of “diagnosis of sepsis” can be achieved through randomized controlled trials of any interventions to confirm improvement in outcomes.

## Disclosure

Conflict of Interest: None declared.
